# Supplementary material for: Understanding Estimations of Magnitudes: An fMRI Investigation
Source: Brain Sci. 2022 Jan 12;12(1):104. doi: 10.3390/brainsci12010104 (PMC8774251; doi:10.3390/brainsci12010104)
Supplement: Supplementary file 1 [file brainsci-12-00104-s001.zip › brainsci-1524569-supplementary.pdf]

Table S1. Descriptive Statistic of the fMRI Task, Behavioral Results.

**Category**	**Knowledge**		**Quantity**		**Time**		**Weight**	
	**Average**	**S.T.D**	**Average**	**S.T.D**	**Average**	**S.T.D**	**Average**	**S.T.D**
Accuracy	82%	4.3%	62%	16%	45%	13%	52%	19%
Absolute distance	0.2	.066	0.43	.20	0.62	.041	0.52	.23
RTs	1589	217	1806	149	1805	152	1824	167
